# Ionically crosslinked cellulose nanocrystals by metal nitrates for the preparation of stable emulsions with tunable interface properties

**DOI:** 10.1038/s41598-023-48703-3

**Published:** 2023-12-07

**Authors:** Joseph Batta-Mpouma, Gurshagan Kandhola, Jin-Woo Kim

**Affiliations:** 1 Bio/Nano Technology Group, Institute for Nanoscience and Engineering, University of Arkansas, Fayetteville, AR 72701 USA; 2 Materials Science and Engineering Program, University of Arkansas, Fayetteville, AR 72701 USA; 3 Department of Biological and Agricultural Engineering, University of Arkansas, Fayetteville, AR 72701 USA

**Keywords:** Materials science, Nanoscience and technology

## Abstract

Biologically extracted cellulose nanocrystals (CNCs) are rod-like and amphiphilic materials with surface-exposed (hydrophilic sites) and hidden (hydrophobic sites) hydroxyl groups. These physicochemical characteristics make CNCs suitable for use as emulsifying agents to stabilize emulsions. Stable oil-in-water emulsions, using sulfated (i.e., –${{\text{SO}}}_{3}^{-}$) CNCs that were ionically crosslinked with alkaline-earth (i.e., ${{\text{Mg}}}^{2+}$) or transition-d-block (i.e., ${{\text{Zn}}}^{2+}$) metal cations, were developed without the use of any synthetic surfactants or prior functionalization of pure CNCs with hydrophobic molecules. Various emulsion surface properties such as interfacial tension, surface charge, surface chemistry, as well as rheology were characterized. Ionically crosslinked CNCs (iCNCs) adsorbed at the interface of an oil and water and fortified the emulsion droplets (5–30 µm) against coalescence by lowering the interfacial tension from 65 mN/m (i.e., pure CNC mixture with oil) to 25 mN/m (i.e., iCNC mixture with oil) and reducing zeta potential with surface charge values (–30 mV to –10 mV), ideal to maintain droplet layer assembly at the water–oil interface. This study provided an alternative approach to achieve particle-stabilized and surfactant-free emulsions by using divalent metal nitrates to develop “clean” emulsion-based technologies for applications in many industries from agriculture to food to pharmaceuticals.

## Introduction

Mixtures of non-miscible liquids form emulsions with the addition of surface-active agents such as polymer-based or nanoparticle-based surfactants^1–3^. By virtue of their amphiphilic nature, surfactants create uniform dispersions of the two insoluble liquids by lowering the surface tension of the mixture and forming emulsions^1^. Surfactants are made of end groups whose one part is hydrophilic and the other hydrophobic, consequently creating a "bridge" between the two immiscible phases. Emulsions are valuable for many applications and play important roles in many industries, from agriculture to food to pharmaceuticals^4,5^. While emulsion technologies have been used for several years, there is still plenty of room to learn about emulsion science.

Despite the immense number of emulsion systems, producing stable emulsion and characterizing their chemistry and structure has remained a challenge^6–8^. One of the drawbacks of polymer surfactant-based emulsions are their environmental toxicity and poor biodegradability^9^. To overcome that challenge, Pickering emulsions (PEs) that use colloidal solid particles as surface active agents have been pursued over decades^10–12^. In principle, the stability of a PE depends on particle wettability (i.e., hydrophobicity or hydrophilicity at the surface of colloids)^13–15^. Based on their wettability, PE will form an oil in water (o/w) or a water in oil (w/o) emulsion for a hydrophilic or hydrophobic surface, respectively^16–18^. Pioneering work on Pickering stabilization of emulsions have mostly been done using inorganic materials such as silica^19,20^, calcium carbonate^21,22^, graphite^23,24^, and clays like montmorillonite^25,26^ and laponites^27,28^. However, these particle-like materials are usually non-surface-active and easily aggregate, requiring further complex modification processes to make their surface hydrophilic or hydrophobic, or involving the addition of hazardous co-surfactants that increase the toxicity of the emulsion^29^.

Many polymer alternatives have been explored to develop PEs from bio-based sources, such as lignin, cellulose, starch, and proteins, and these materials do not require any hazardous co-surfactants and/or intensive surface modifications^29^. Among various emulsion stabilizers, cellulosic materials are the most desirable due to their physicochemical characteristics and the abundant supply of agricultural and forestry residues as well as waste generated in the paper mill industry, with a production of nearly 1.5 × 10^12^ tons per year^30,31^. Cellulose structures are made of crystalline and amorphous regions^32^. In order to extract the crystalline part, namely cellulose nanocrystals (CNCs), controlled acid hydrolysis and mechanical treatments are performed on microfibrillar cellulose to remove the amorphous regions^32^.

Depending on their source, physical nature of CNCs shows structures with rod shape and size in the range value of 70–500 nm in length, and 2–20 nm in width^32,33^. Moreover, when cellulose is hydrolyzed by sulfuric acid, CNCs bear sulfated and hydroxyl groups at their surface, enabling the control of their physicochemical properties and providing reactive sites for ionic (O_3_SO^*−*^) or covalent (OH) crosslinking^34–36^. The presence of sulfated (O_3_SO^−^) and OH groups at the surface of CNC defines the chemical nature and reactivity of pure CNC extracted from sulfuric acid hydrolysis. The C6 carbon holds the O_3_SO^−^ and carbons at the C2 and C3 positions hold the OH group^37^. Owing to their surface and physicochemical properties, colloidal CNCs have been explored and found suitable to stabilize PEs^38,39^. Although colloidal CNCs are polar materials that are dispersible in water, their amphiphilic character has also been regarded as the driving factor in making stable PEs^39,40^. In a study^41^, while examining the effect of sulfated CNCs and their wettability on PE formation, the surface activity of CNC was found hydrophobic along the (2 0 0) plane edge, while hydrophilic along the (0 1 0), (1 1 0) and (1 $\overline{1 }$ 0) facets. CNCs were shown to have a structural anisotropy that could be linked to their amphiphilic behavior, implying that colloidal CNCs have both non-polar (hydrophobic) and water polar (hydrophilic) surfaces^42^. A modified strategy yielded amphiphilic CNCs with higher degree of hydrophobicity by functionalizing its end-group by polystyrene (PS), showing that the composite CNC-PS was highly effective in making emulsions of toluene and hexane than colloidal CNCs from sulfuric acid hydrolysis^39^.

In previous studies, particle-stabilized emulsions were prepared by functionalizing CNCs with hydrophobic materials^13,39^, using fully OH covered CNCs synthesized by HCl hydrolysis^41,43^, or screening surface charges with monovalent salts (NaCl, KCl, or HCl)^41^. Although these methods have shown promise for colloidal CNCs to be good PE stabilizers, there has not been any study using metal nitrates of Mg^2+^ and Zn^2+^, to control the repulsive forces at the CNC-CNC interface during the formation of emulsion droplets. While most salts help partition more emulsifiers to the interface and help reduce the interfacial tension, divalent cations are expected to work better due to their superior valence; they result in stronger ionic crosslinking with CNCs and enhanced emulsion stability.

In this study, a straightforward route was adopted to prepare ionically crosslinked CNCs (iCNCs) for emulsions with tunable surface properties. The amphiphilic character of iCNCs was exploited to facilitate the adhesion of particles at the o/w interface to enhance the formation and stability of emulsions without the use of petrochemical polymers as surfactants. There has been interest in the literature to study the effect of salt in emulsion models, but the understanding of how these salts solutions impact CNC-stabilized emulsions is still limited. Therefore, the focus of this work was to identify the physicochemical characteristics of iCNCs that play a role in the production of stable o/w emulsions and demonstrate the effect of sulfated CNCs and divalent salt concentrations on the formation mechanism of PEs for their practical uses.

Moreover, as charge distribution with the use of monovalent salts has been demonstrated as a problematic parameter in the formation of PEs^41^, the effect of divalent salts, such as alkaline (Mg(NO_3_)_2_) and transition metal (Zn(NO_3_)_2_) cations, was investigated. In agriculture, for example, nitrates have been used as a good source of fertilizer, and they are less corrosive than chlorides. Assuming drinking or regular water is used for agriculture purposes (e.g., preparing for tank mixes), there is a higher risk of built-up or precipitation arising from chlorides than nitrates; for example, MgCl_2_ reacts with AgNO_3_ [mostly found in water] solution to give precipitate of AgCl, whereas Mg(NO_3_)_2_ does not react with AgNO_3_ solution to give a precipitate. Theses emulsions will have advantages when used in food and pharmaceutical products as well because of two reasons: (1) nutritional benefits associated with the intake of Mg and Zn, and (2) overtime NaCl that may be a concern for individuals on physician prescribed “no salt diets” even when consumed at low doses. In this study, oleic acid (OA) was used as a model oil owing to its low (i.e., 1.0) hydrophilic-lipophilic balance (HLB) and exposed carboxylic groups in the aqueous media, inducing the adsorption to amphiphilic colloids at the interface^44^. A low HLB number implies a strong oil affinity, which is an important characteristic for the formation of an o/w emulsion. Additionally, not only OA is a natural fatty acid found in various animal and vegetable sources, but it is also used as a pharmaceutical excipient and an emulsifying agent in droplet products. Understanding the composition and the preparation process of emulsions stabilized by ionically crosslinking CNC with divalent metal nitrates and tunability in terms of surface chemistry and charge, as well as rheology and interfacial tension, would benefit the development of advanced bio-based emulsions to serve many industries such as agriculture, food, and pharmaceutical, to name a few.

## Results and discussion

### Emulsion formation and droplet size measurement

**Figure 1:**
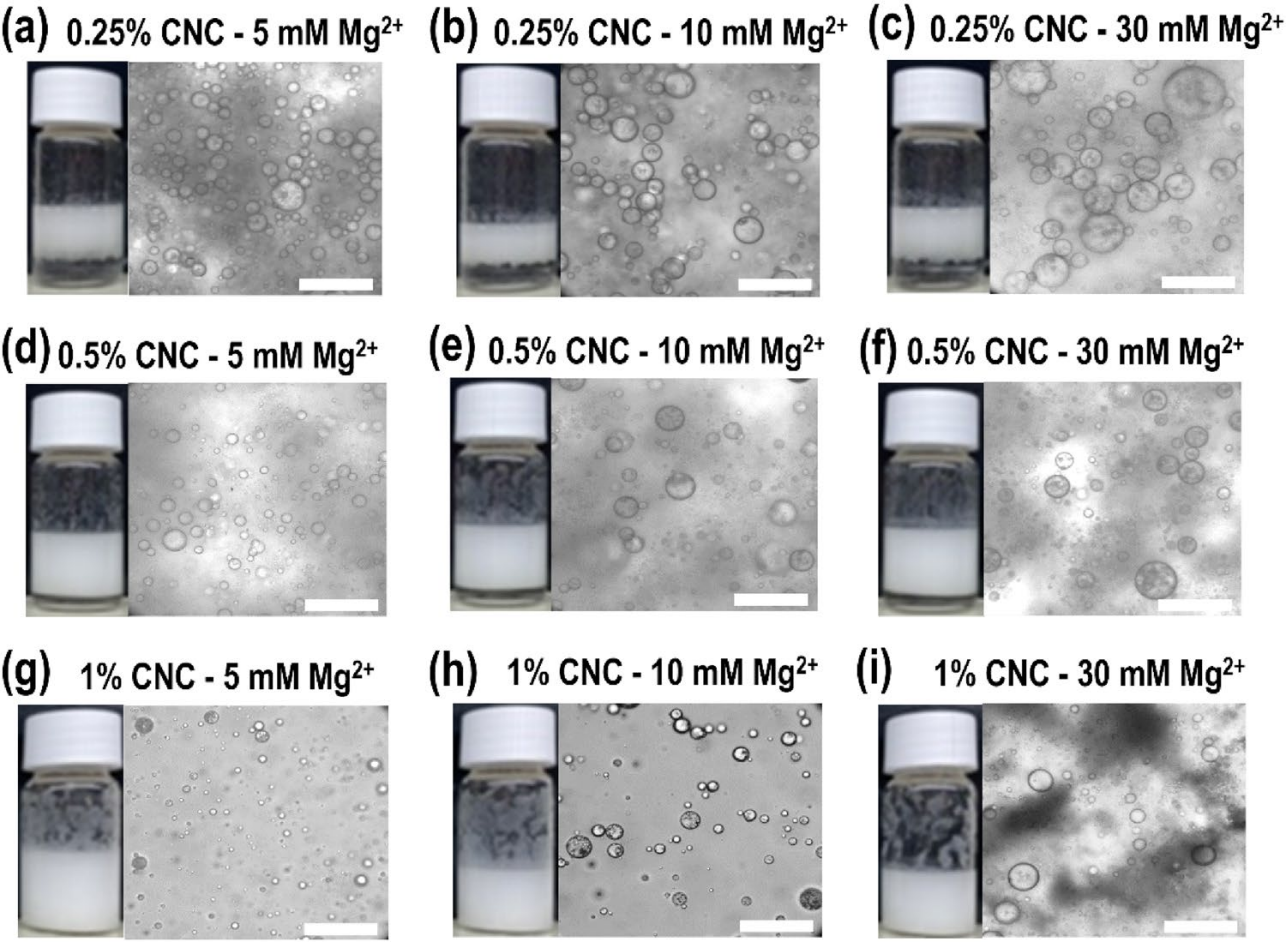
Optical micrographs of tunable CNC emulsion droplets 24 h post preparation at varied concentrations of CNCs and Mg(NO_3_)_2_. Scale bars, 200 μm.

**Figure 2:**
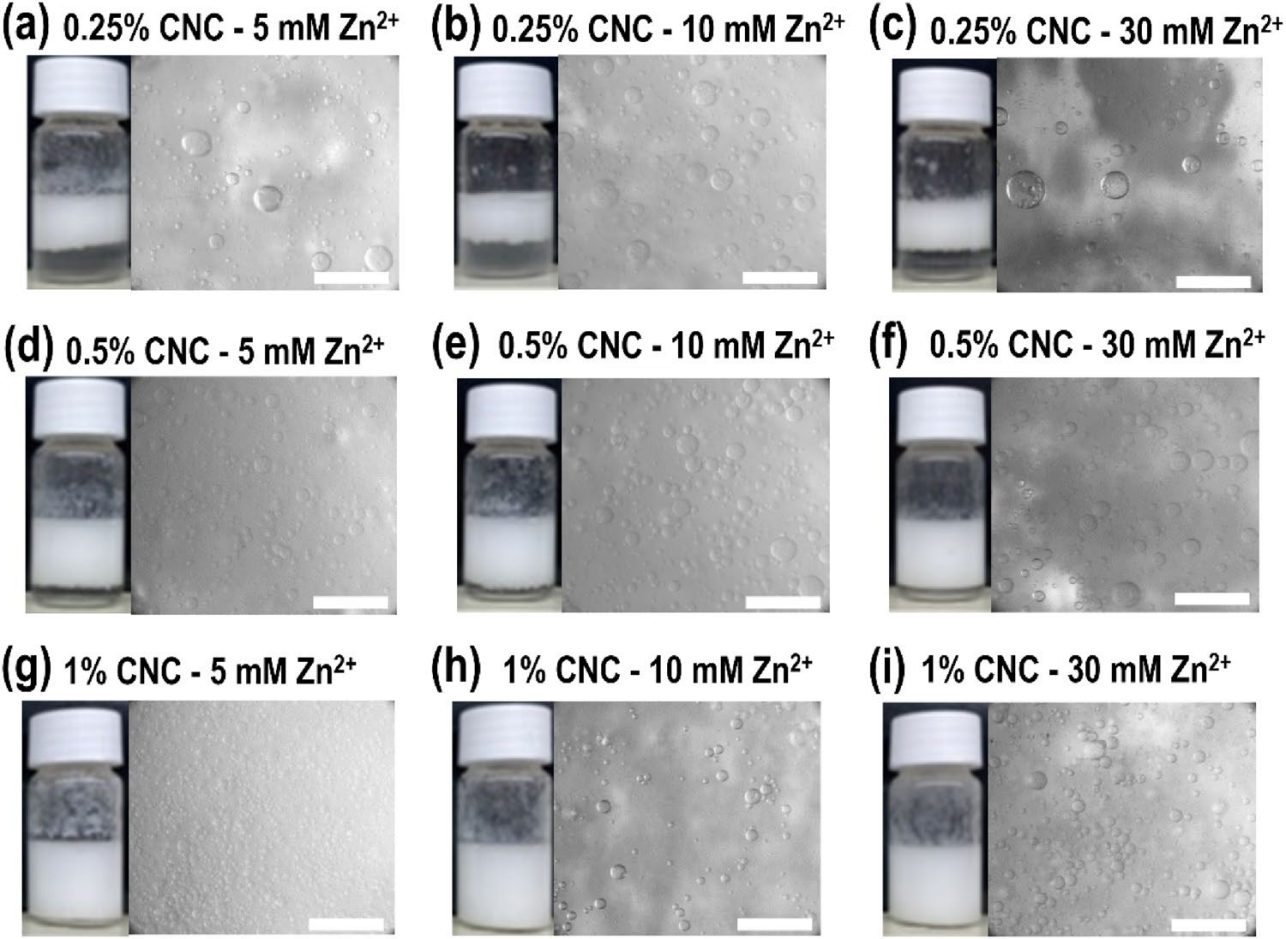
Optical micrographs of tunable CNC emulsion droplets 24 h post preparation at varied concentrations of CNCs and Zn(NO_3_)_2_. Scale bars, 200 μm.

**Figure 3:**
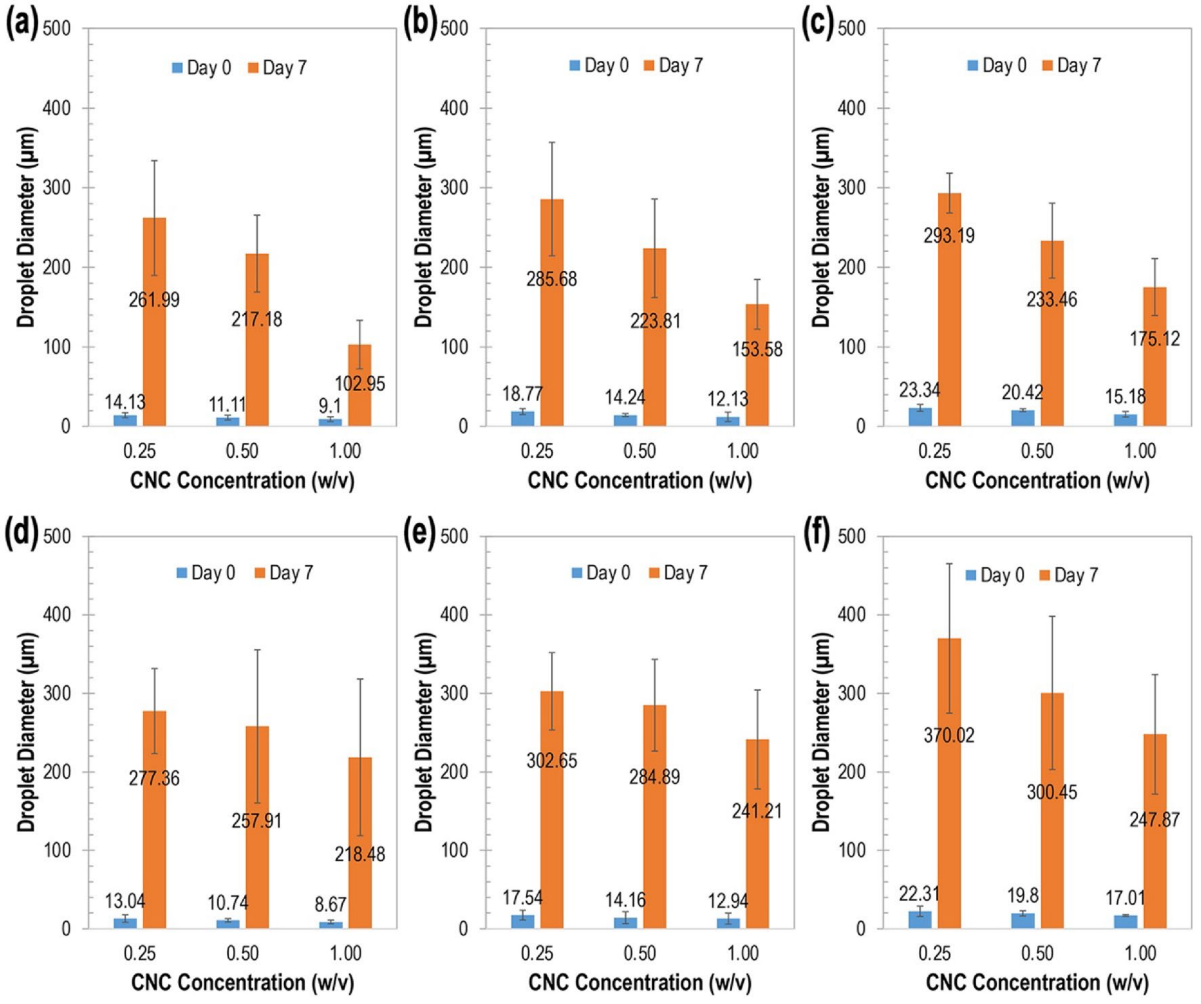
Stability of emulsion droplets over time. (**a–c**) Ionically crosslinked CNC (iCNC) at varied concentrations of Mg^2+^: (**a**) 5 mM, (**b**) 10 mM, and (**c**) 30 mM. (**d–f**) iCNC at varied concentrations of Zn^2+^: (**d**) 5 mM, (**e**) 10 mM, and (**f**) 30 mM.

Micrographs were obtained to determine size distribution and visualize the microstructure of emulsion droplets as displayed in Figs. 1 and 2. While it was observed that the presence of iCNC by divalent cations does not prevent the formation of emulsions, the dissociation of droplet was affected over time as the emulsion is thermodynamically instable (Fig. 3). For measurements done within 24 h and at around one week (see Supplementary Figs. S1–S4), Fig. 3 showed droplet size diameter was found in the value range from 5 to 30 µm (i.e., day 0) and from 100 to 450 µm (i.e., day 7), respectively, indicating that emulsions stabilized by iCNCs met the eligibility requirements of PEs as the size of droplets in the emulsion of this work was on average equal or higher than 10 µm^45^. It was observed that over a period of one week, droplet diameter increased in size, suggesting that the ionically crosslinked CNC at the layer of each droplet dissociated at the CNC–CNC interface.

When CNC concentrations were kept constant, droplet sizes increased (*p* < 0.05) as salt concentration was increased, suggesting the accumulation of crystals at the o/w interface is minimal. But when salt concentrations were kept constant, droplet size decreased (*p* < 0.05) with increasing CNC concentration. Emulsions made with colloids were more stable when increasing particle volume fractions and concentrations were employed due to higher extent of particle bridging as previously reported^38^. A similar behavior of the creaming process was observed as CNC concentrations increased from 0.25 to 1% (w/v) in our study (Figs. 1 and 2).

Micrographs displayed uniform spherical droplets for all samples with minimal to no coalescence in all emulsions, suggesting that iCNCs facilitated the mixture of an oil in water by forming a stable emulsion. When the emulsion was made with CNCs without the use of divalent cations, the droplets disassociated within minutes (see Supplementary Fig. S5), suggesting that ionic crosslinkers participate in the structural stabilization of the outer layer of emulsion droplets. On the one hand, when CNC concentrations increased from 0.25 to 0.5% to 1% (w/v), we observed an incremental milking and creaming homogeneity of the emulsion from a partial (Figs. 1a–c and 2a–c) to moderate (Figs. 1d–f, 2d–f) to full miscibility (i.e., no observable serum at the bottom of the vial containing the dispersion (Figs. 1g–i and 2g–i) of the two phases. The same trend was observed for measurements done on one week old emulsions (see Supplementary Figs. S3 and S4).

On the other hand, emulsions made of two non-miscible liquids, in which iCNCs were dispersed in oil, led to the formation of stable micron size droplets. This observation allows to deduce that iCNCs acted as emulsifying agents and chemical bridges between the two liquids in the dispersion. As particles are more strongly held at the interface owing to ionic crosslinking, they are prone to be held into the continuous phase during a bridging occurrence. Also, higher concentrations of colloidal particles would further speed up the assembly of the droplet, leading to a more stable emulsion. This suggests that bridging is affected not only by surface-active compounds that adsorb at the boundary between an oil and water, but also by particle interactions and their packing at the surface of the colloids.

### Effect of surface charge

**Figure 4:**
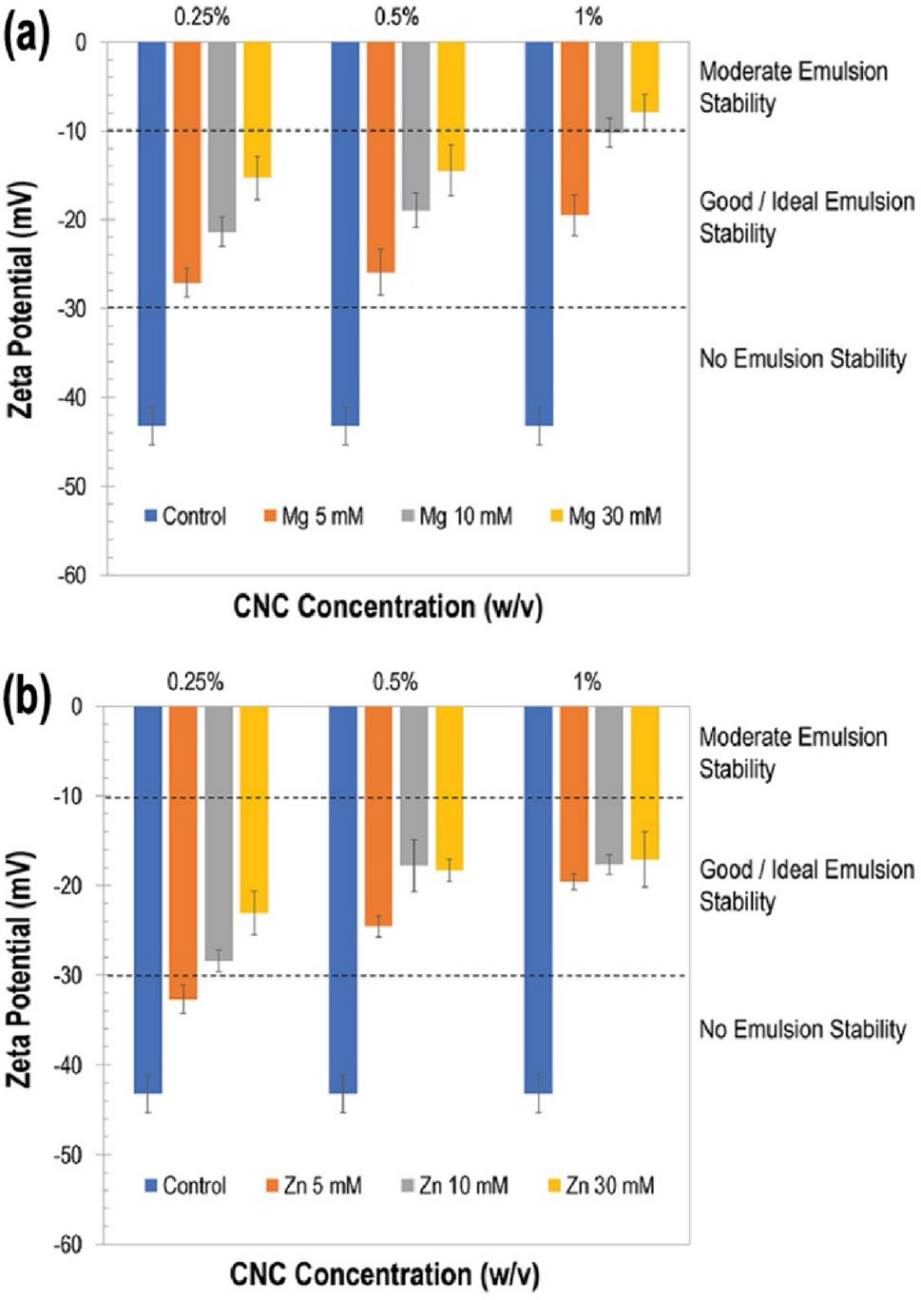
Evaluation of the zeta potential of emulsion droplets as a function of ionically crosslinked CNCs (iCNCs) at varied concentrations of (**a**) Mg(NO_3_)_2_ and (**b**) Zn(NO_3_)_2_ to show the effect of surface charge on the formation of droplets.

Zeta potential (ZP) was used to evaluate the effect of ionic crosslinking on emulsion stability by determining the magnitude of charge on the surface of emulsion droplets (Fig. 4). ZP values were determined close to neutral values of pH 6. The control emulsions (i.e., stabilized by pure CNCs without any salt or ionic crosslinkers added) presented the highest negative ZP (–43.26 ± 2.11 mV) attributable to the layer of deprotonated or sulfonated (^−^OSO_3_) CNCs at the droplet interface. Such a high ZP leads to electrostatic repulsive forces (between adjacent CNCs at the interface) having an overpowering effect, resulting in the CNC layer breaking up and emulsion droplets coalescing over time (see Supplemental Fig. S5). This suggests that sulfonated CNCs alone are not good candidates for long-term stability of Pickering emulsions.

When positively charged, divalent salts were added to the network of negatively charged CNCs, a decrease in ZP values was observed, indicating reduction of effective charge on the surface of emulsion droplets due to ionic crosslinking between adjacent CNCs via cations. At each CNC concentration [i.e., 0.25%, 0.5%, or 1% (w/v)], the ZP of emulsions containing bimetallic salts was lower than that of the respective control emulsions owing to enhanced van der Waals forces (dipole–dipole interactions between cations and CNCs as well as H-bonding between adjacent CNCs). In general, for all combinations of CNC and salt concentrations tested in this study (except 1% CNC with 10 mM or 30 mM Mg and 0.25% CNC with 5 mM Zn), ZP ranged between –30 and –10 mV, indicating improved emulsion stability with ionically crosslinked CNCs.

Moreover, it was noticed that, as salt concentration increases (for both Mg and Zn), surface charge on the droplet or ZP of the emulsion decreases. This is because interparticle electrostatic repulsive forces between CNC particles decrease and attractive van der Waals forces increase, leading to the formation of a more stable layer of crosslinked CNCs at the o/w interface that prevent droplet coalescence in the long term. Consequently, the presence of salt decreases the effective surface charge at the droplet layer by decreasing the electric double layer thickness and favoring the extent of interparticle crosslinking, as shown in Fig. 4.

In a previous study, similar observations were made by evaluating the effect of salt concentration on the bridging of CNCs^46^. This study showed that the CNC bridging increases as NaCl concentration gradually increases, due to the electrostatic screening effect from the cation counter ion. We observed that the salt concentration at 30 mM promoted minimal flocculation levels in the emulsion, indicating the effect of ionic strength in favoring the formation of crosslinks between negatively charged CNCs by positively charged metal crosslinkers. Samples made of CNCs in salts before their mixture with the oil showed comparable lowering of ZP values, indicating iCNCs are responsible for the formation of stable emulsions (see Supplementary Fig. S6).

**Figure 5:**
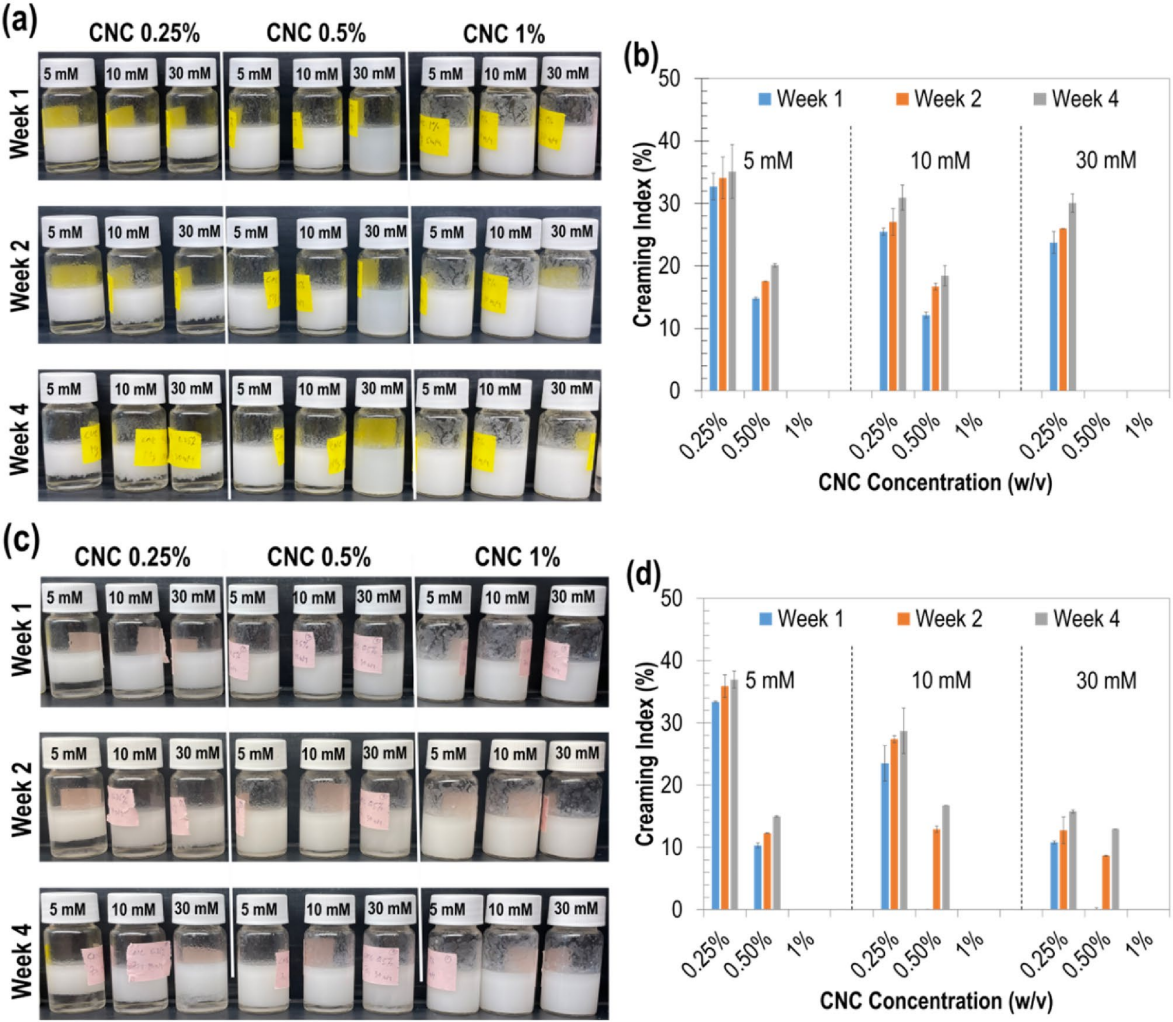
Emulsion stability over 4 weeks: (**a,c**) visual observations and (**b,d**) creaming indices of iCNC-stabilized emulsions at varied concentrations of Mg(NO_3_)_2_ (**a,b**) and Zn(NO_3_)_2_ (**c,d**).

Overall, mid-range ZP values (between –10 and –30 mV) were found to be ideal for emulsion stability. In this ZP region, charges at the CNC interface are neither too high (i.e., around –40 mV as for control emulsions containing pure CNCs) to cause electrostatic repulsion, nor too low (i.e., less than –10 mV) to cause droplet aggregation and coalescence. This observation was consistent across all concentrations (w/v) of iCNCs. The lowest values for surface charges were obtained for 1% CNCs using 30 mM Mg(NO_3_)_2_ salt (i.e., ZP = –7.9 ± 1.9 mV) (Fig. 5a), and 30 mM Zn(NO_3_)_2_ salt (i.e., ZP = –20.1 ± 3.08 mV) (Fig. 5b). For these optimal charges on emulsion droplets, moderate to good (i.e., ideal) emulsion stabilities were observed.

### Emulsion stability

Depending on the concentration of iCNCs, the emulsions displayed varied stability and formed either a milky phase on the top and a water phase at the bottom ($CI$ < 100%) or a single creaming layer ($CI$ = 100%), owing to the difference in surface charge at the droplet layer (Fig. 4). This observation was aligned with results regarding the effect of surface charge (Fig. 4). As the concentrations of CNC and salt increase, the surface charge decreases with the formation of PEs with varied stability (Fig. 4). With ZP values decreasing with increasing ionic crosslinker concentrations, it was suggested that Mg^2+^ and Zn^2+^ had different affinities when crosslinking sulfated CNCs with negative charges. This reactivity was due in part to the difference in electronegativity of the ionic crosslinkers used in this work. For a chemical bonding (i.e., ionic bond) to take place, the energy required to permanently share an electron from one atom to stabilize another depends on the charge density of interacting atoms. Similar ionic bonding between colloidal CNCs and multivalent ions of opposite charge to form crosslinking points by electrostatic interaction was reported previously^47^.

The amount of energy required for this transfer to take place depends on the valence (i.e., the number of electrons available in the outermost shell of an atom to be transferred) and their electronegativity. With elements of the same valence, the higher the electronegativity of an element, the higher its tendency to form ionic bonds with other elements. Based on the periodic table in relation to electronegativity values, Mg has an electronegativity of 1.3 while that of Zn is 1.7. The ionic bond between ${{\text{Mg}}}^{2+}$ or ${{\text{Zn}}}^{2+}$ and –${{\text{SO}}}_{3}^{-}$ (sulfate) occurs at the negatively charged oxygen of the sulfate group on CNCs. The electronegativity superiority of Mg over Zn was hypothesized to be the reason behind its better propensity to ionically crosslink CNCs. Because Mg loses its electrons more easily than Zn, a nucleophilic group (sulfate in this case) containing oxygen reacts more vigorously with Mg than with Zn. This was reflected in surface charge and interfacial tension (IFT) results, i.e., relatively lower ZP as well as lower IFT for Mg when compared with Zn at similar concentrations.

Overall, the addition of any salt caused the reduction of charges at the droplet layer of the iCNC-stabilized emulsion by linking divalent ions to sulfated CNC and accumulating the crosslinked allomorph at the surfaces of droplets, confirming our previous observation made from droplet size distribution (Figs. 1 and 2). As the ZP describes the charge difference between water and the oil bound to the dispersed droplet, there seems to be a benefit with the bridging effect during the formation of stable emulsions with solid particles like iCNCs. Many emulsion technologies, especially food emulsions, have taken advantage of this aspect as it facilitates the stability of emulsions^9^.

### Creaming index (CI)

Observation of iCNC-based emulsions prepared with different concentrations of CNCs and salts, as well as *CI* percent values are shown in Fig. 5. The prepared iCNC-based emulsions were monitored at room temperature (22 °C) for four weeks and the stability was determined by $CI$ calculation and visual observation of creaming over time (Fig. 5a, c). The results showed that $CI$ decreased as CNC and salt concentrations increased, and that all values were around or below 40% as previously reported^48^. As iCNC concentration increased, the level of creaming was generally lower in the emulsions, implying stronger emulsion stabilization. The higher the $CI$, the lower is the emulsion stability. Previous studies using CNCs have reported similar observations for the effect of creaming on the formation of stable emulsions^48,49^. Emulsions prepared with 1% iCNC had the lowest $CI$ for 30 mM of both salts (i.e., Mg(NO_2_)_3_ and Zn(NO_2_)_3_), hence displaying better stability compared to emulsions prepared with 0.25% iCNC and 5 mM salt, which displayed the highest $CI$ (*p* < 0.05). However, for the same salt and CNC concentrations, $CI$ did not significantly change over time from week one to week four after preparation (Fig. 5b, d).

Another point to note is that, when emulsification was done with decreasing CNC and salt concentrations, $CI$ had an increasing value trend. This suggests that the network structure via particle–particle interactions provided minimal CNC packing, thus resulting in the formation of thinner outer layers at the droplet surface that can be disrupted easily. Decreasing $CI$ due to increase in CNC and salt concentrations revealed that ionic crosslinking favored the formation of a stronger protecting area around the oil droplet that protects them from coalescence. This suggested that the bonding between CNCs confers a denser structural layer that maintains the stability of the PE over time without a noticeable disruption.

### Effect of interfacial tension (IFT)

**Figure 6:**
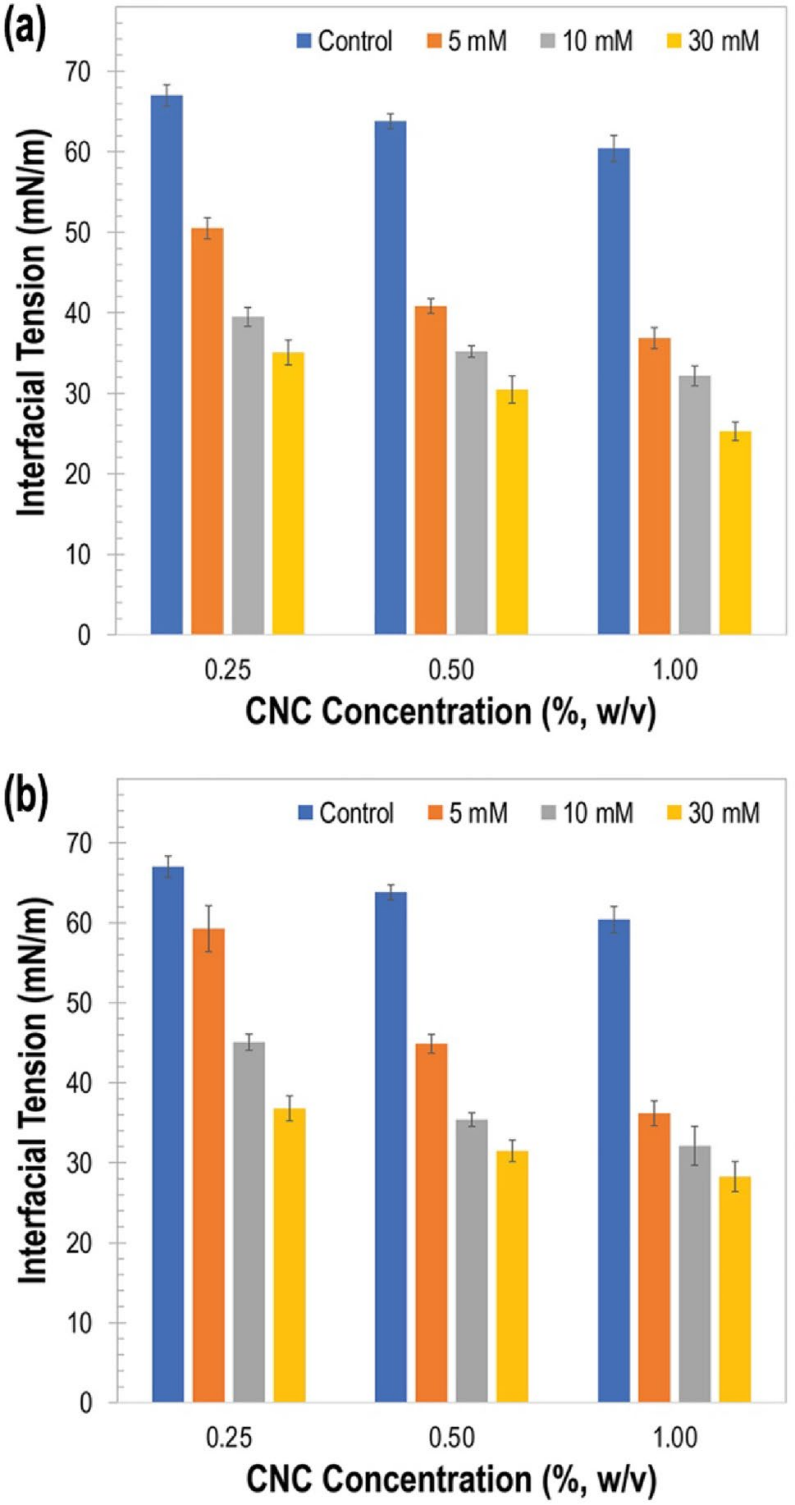
Interfacial tension of emulsions as a function of ionically crosslinked CNC (iCNC) at varied concentrations of (**a**) Mg(NO_3_)_2_ and (**b**) Zn(NO_3_)_2_ to show the effect of particle adsorption at the o/w interface.

IFT behavior of the emulsions was evaluated at varied CNC and salt concentrations as shown in Fig. 6. It was observed that as concentration of the CNC increased, IFT decreased, suggesting that pure CNC had the ability to lower the IFT of the emulsion. CNCs, as synthesized by acid hydrolysis, are in aqueous phase. At a low CNC concentration [i.e., 0.25% (w/v)], emulsion stabilized by the pure CNC showed an IFT of 67.01 ± 1.42 mN/m, lower than that of water (i.e., 72.5–73 mN/m). When CNC concentration was increased to 1% (w/v), IFT significantly decreased (*p* < 0.05) to an IFT value of 60.14 ± 1.64 mN/m. From these observations, it was deduced that pure CNCs have an inherent ability to lower the IFT at the o/w interface via adsorption of colloidal particles at the surface.

As described above for the effect of surface charge, it was shown that the reduction of ZP was facilitated by salt addition, which favored the formation of a stable emulsion. That observation aligned with the interfacial behavior of emulsions as close packing of CNCs at the o/w interface, which was facilitated by ionic crosslinking, helped reduce IFT even further when compared to pure CNCs. It was observed that by increasing the concentration of salt, the IFT of the emulsion further decreased and the trend remained the same across all CNC concentrations. When 0.25% iCNCs were used, IFT significantly decreased from 50.51 ± 1.31 mN/m to 39.49 ± 1.17 mN/m to 35.07 ± 1.55 mN/m as Mg(NO_3_)_2_ concentration increased (Fig. 6a). The same trend was observed for 0.25% iCNC when the concentration of Zn(NO_3_)_2_ increased from 5 to 10 mM to 30 mM, with decreasing IFT values from 59.26 ± 2.86 mM/m to 45.07 ± 1.02 mN/m to 36.81 ± 1.58 mN/m (Fig. 6b).

In the same manner, when the highest concentration (1% CNC) was used in their ionically crosslinked form, IFT further reduced from 36.84 ± 1.31 mN/m to 32.18 ± 1.22 mN/m to 25.27 ± 1.16 mN/m by increasing Mg(NO_3_)_2_ salt concentration from 5 to 10 mM to 30 mM (Fig. 6a). A similar trend was observed by increasing Zn(NO_3_)_2_ salt concentrations, as results showed decreasing IFT values from 36.19 ± 1.57 mN/m to 32.10 ± 2.43 mN/m to 28.27 ± 1.89 mN/m (Fig. 6b). These observations confirmed that ionic crosslinking led to enhanced CNC adsorption at the emulsion droplet layer, resulting in reduced IFT and enhanced emulsion stability, an effect that was more pronounced at higher salt and CNC concentrations. Although CNC-stabilized emulsions were similar to conventional surfactant-stabilized emulsions, their mechanism to stabilize emulsions using ionic crosslinking was different from typical ones. As CNC is an amphiphilic material, increased crosslinking by salt concentration would preferably favor the mixture of CNC with the oil phase as sulfated group on the nanocrystal are shielded.

Overall, it was hypothesized that, as the surface charge of CNCs decreases, the ionic strength increases, thus reducing repulsive forces between CNC particles and augmenting particle assembly at the interface. A similar observation was reported previously while studying the effect of polymer-grafted lignin nanoparticles on the tunability of PEs^50^. As iCNCs adsorbed at the o/w interlayer and created a mechanical barrier that protected the emulsion droplets against destabilization, it was interpreted that the properties of amphiphilic CNCs are responsible to prevent droplet aggregation, lower IFT, and improve emulsion stability.

### Effect of surface chemistry

**Figure 7:**
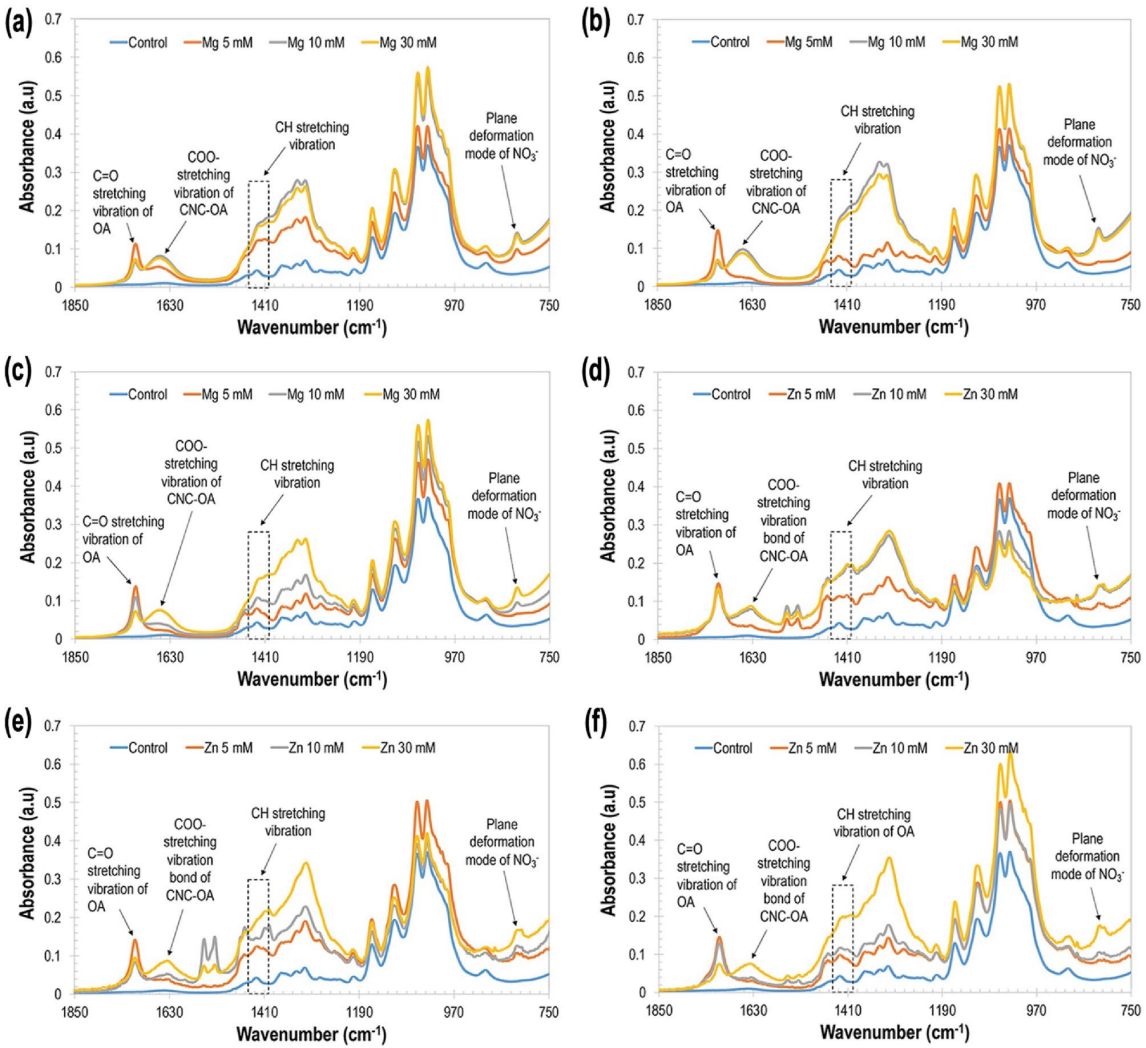
Fourier transform infrared (FT-IR) as a function of wavenumber: (**a**) 0.25% CNC (w/v) and Mg(NO_3_)_2_, (**b**) 0.5% CNC and Mg(NO_3_)_2_, (**c**) 1% CNC and Mg(NO_3_)_2_, (**d**) 0.25% CNC and Zn(NO_3_)_2_, (**e**) 0.5% CNC and Zn(NO_3_)_2_, and (**f**) 1% CNC and Zn(NO_3_)_2_.

Fourier transform infrared (FTIR) spectra of the emulsions prepared with varied concentrations of CNCs and salt types are shown in Fig. 7 for infrared (IR) spectra from 1850 to 750 cm^−1^. The full IR spectra from 4000 to 750 cm^−1^ displaying typical peaks for pure and modified CNCs are provided in Supplementary Fig. S7. When different components (i.e., iCNCs in water phase and oleic acid as oil phase) are mixed, physical blending as well as chemical interactions occur and the changes in their spectral intensity peaks can be evaluated^51^.

Band spectra of pure CNCs from 3600 to 3000 cm^−1^ were known for the stretching of bonded O–H, 3000–2800 cm^−1^ to C–H stretching, 1400–1300 cm^−1^ to free O–H, 1414 cm^−1^ to asymmetric stretching of C–O–H and 1060 cm^−1^ to bending of C–O–C, and wavenumber peak at 807 cm^−1^ was recognized by the presence of a sulfate ester, revealed by the C–O–S vibration. The increase in peak intensity around 1650 cm^−1^ was due to the interaction between the sulfate group (^−^OSO_3_) on CNCs and bimetallic salts. The vibration of ^−^OSO_3_ was recognized at 1207 cm^−1^, suggesting the presence of ionic bond interaction and complexation between negatively charged sulfated CNCs. The weak peak intensity around 820 cm^−1^ was assigned to the out of plane deformation mode of nitrate ions (NO_3_^−^)^52^, signifying the use of Zn and Mg nitrate salts for the ionic crosslinking process (Fig. 7). In general, increase or shift in the peak position is due to the interaction of CNC and salts, indicating the complexation of sulfated CNCs by bimetallic cationic linkers.

### Effect of viscosity

**Figure 8:**
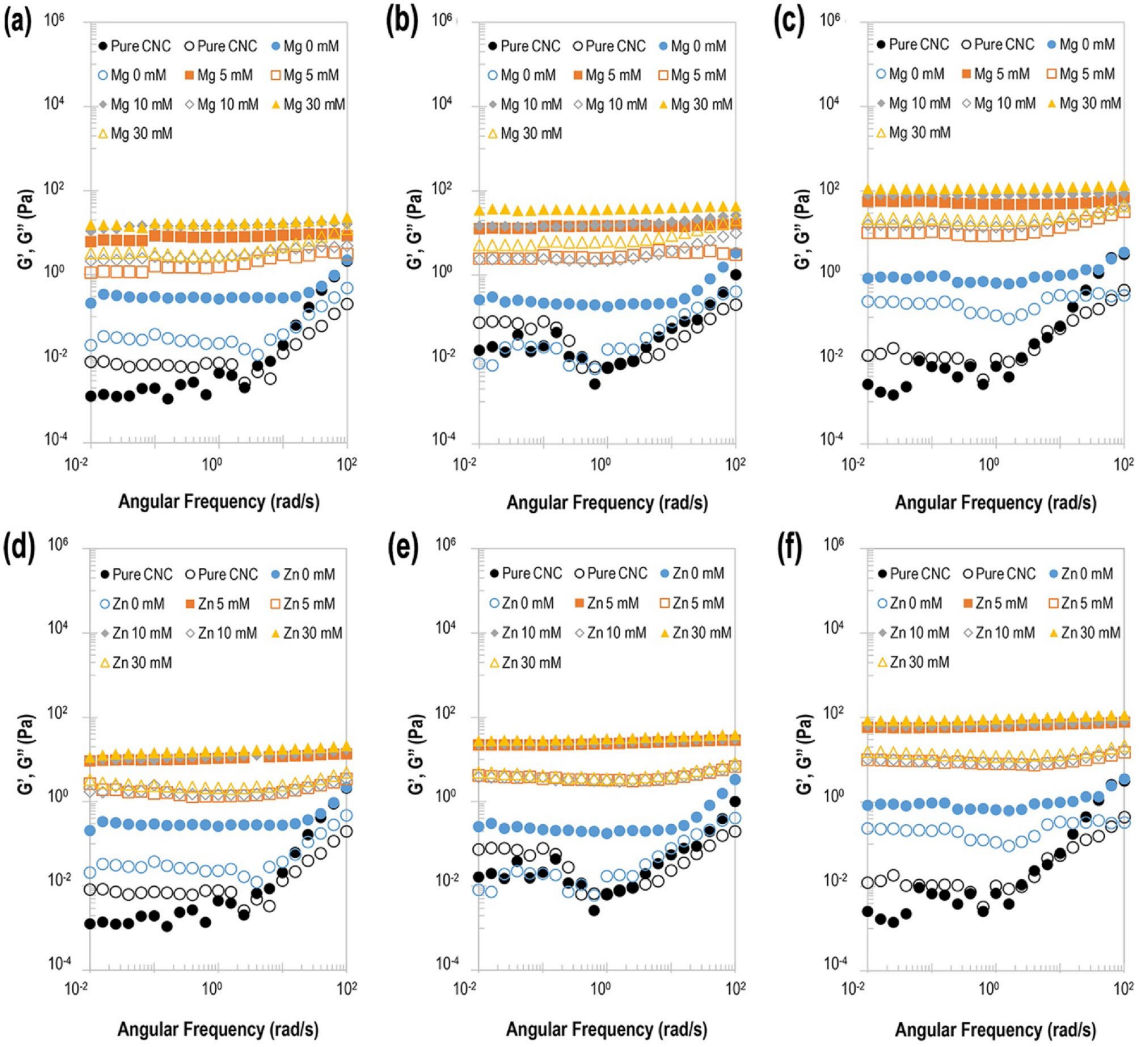
Dynamic moduli at varied concentrations of CNCs and divalent salts. (**a–c**) Mg(NO_3_)_2_: (**a**) 0.25% (w/v), (**b**) 0.5% (w/v), and (**c**) 1% (w/v) of CNCs. (**d–f**) Zn(NO_3_)_2_: (**d**) 0.25% (w/v), (**e**) 0.5% (w/v), and (**f**) 1% (w/v) of CNCs. Open symbols: storage moduli (${G}{\prime}$). Closed symbols: loss moduli (${G}^{{\prime}{\prime}}$).

The progression of the dynamic moduli [storage (G′) and loss moduli (G′′)] was used to evaluate emulsions stabilized by iCNCs (Fig. 8). It was found that the viscoelastic properties of emulsions were mostly dependent on the concentration of CNCs with little effect by salt concentration, leading to both a quantitative change in the absolute values of the linear viscoelastic functions and a qualitative change in the evolution of these functions with frequency. G′ was always higher than G″ with an apparent monotone trend in the tested frequency range of 0.01 rad/s to 100 rad/s when iCNCs were used. This behavior suggests there is a network with a more elastic than viscous behavior because of the occurrence of an extensive bridging process at the CNC-CNC interfaces. G′ and G′′ increase with CNC concentrations appeared to show a plateau for all iCNC-stabilized emulsions to the contrary of pure CNC only (i.e., colloids as synthesized without ionic crosslinking, salt, nor oil) or pure CNC mixed only with oil, which showed no trend (Fig. 8). Not only does this indicate that iCNCs formed microstructures that were less sensitive to oscillatory stress, but also suggests the existence of a more stable internal structure of emulsions formed by iCNCs.

Cox-Merz plots are shown in Supplementary Fig. S8 to evaluate the stability of iCNC-based emulsions through flow curves as a function of CNC and salt concentrations for steady shear rate or oscillation frequency. The Cox-Merz rule has been used and reported to provide information about the microstructure and stabilization of emulsions^53,54^. For an emulsion with structural stability at the outer layer, the Cox–Merz rule stipulates that the complex viscosity (${\eta }^{*}$), at a specific oscillatory frequency ($\omega$), should be nearly equal to apparent viscosity ($\eta$), when $\omega =\dot{\gamma }$.

In a previous study, the implication of pigment additions on the rheological behavior was evaluated, reporting their effect on the microstructural stability of food emulsions^54^. Although both the complex and apparent viscosity values had the same decreasing power-law curve trend (i.e*.*, two flow curves were relatively parallel to each other) (see Supplementary Fig. S8c, f) and the complex viscosity values were higher than those of apparent viscosity for all iCNCs except for control emulsions containing pristine CNCs (see Supplementary Fig. S8a–f), it was observed that all iCNC emulsions do not follow the Cox–Merz rule as the relative viscosities were non-zero values, implying that emulsions were made with colloidal suspension.

It was noticed that the higher was the concentration of CNCs and salt, the lower was the deviation from Cox–Merz rule, suggesting that the microstructural failure provoked by steady shear rate was less pronounced in the case of iCNCs, which were strongly held in place at the droplet outer layer by van der Waals interactions. This observation corroborates our previous description that iCNC-stabilized emulsions were concentration dependent. This observation was aligned with the empirical Cox–Merz rule as reported for other emulsions^54^.

All emulsion compositions displayed the same flow behavior (see Supplementary Fig. S9). The emulsion flow behavior was dependent on the concentration of CNCs, but fluid flow behavior was not compromised. The rheological analyses of the emulsions, through the investigation of their flow behavior in the presence of CNCs [0.25, 0.5, and 1% (w/v)] and ionic crosslinkers (Mg^2+^ and Zn^2+^) at concentrations of 5–30 mM, demonstrated shear thinning behavior with increased viscosity and more stable emulsion droplets as CNC concentration increased (see Supplementary Fig. S9). This evaluation was in agreement with prior observations made with the effect of charge by ZP values and interface tension. Since higher salt concentrations provide lower ZP for stronger outer layer integrity at the droplet interface, iCNCs showed a superior ability to resist droplet aggregation and coalescence.

## Conclusion

Surfactant-free, particle-stabilized emulsions were successfully prepared by ionically crosslinking CNCs with divalent metal nitrate salts of different electronegativity. The effects of CNC and salt concentrations on the stability and the size distribution of emulsion droplets were studied by evaluating physicochemical properties such as surface charge, surface chemistry, interfacial tension, and dynamic moduli.

It was found that increasing the concentration of CNCs in either salt system (i.e., Mg(NO_3_)_2_ or Zn(NO_3_)_2_) improved emulsion stability. The size of the droplets was found to be in the value range from 5 to 30 µm and interfacial tension, as compared to 72.5 mN/m for water, was found to decrease from 67 mN/m (colloidal or pure CNC mixed with oleic acid) to 25 mN/m (iCNC mixed with oleic acid), implying improved surfactant-like characteristics. Moreover, higher concentrations of salt lowered the zeta potential from –40 mV (pure CNCs) to ideal values for the formation of stable emulsions in the range of –30 mV to –10 mV (iCNCs), improving the accumulation of iCNCs due to van der Waals attraction forces between sulfated CNCs and divalent cations, and preventing droplets from collapse. Hence, iCNC-based emulsions have the potential to be useful for a variety of products ranging from pesticide formulations in agriculture and medicinal formulations encapsulating water-insoluble or soluble active ingredients in pharma to natural emulsifying ingredients in food. To generalize the mechanism of emulsifying oil in CNC-crosslinked divalent metal nitrate salts, however, a systematic study of various ratios of water and oil is of intertest.

## Materials and methods

### Preparation of the emulsion-stabilized CNCs

**Figure 9:**
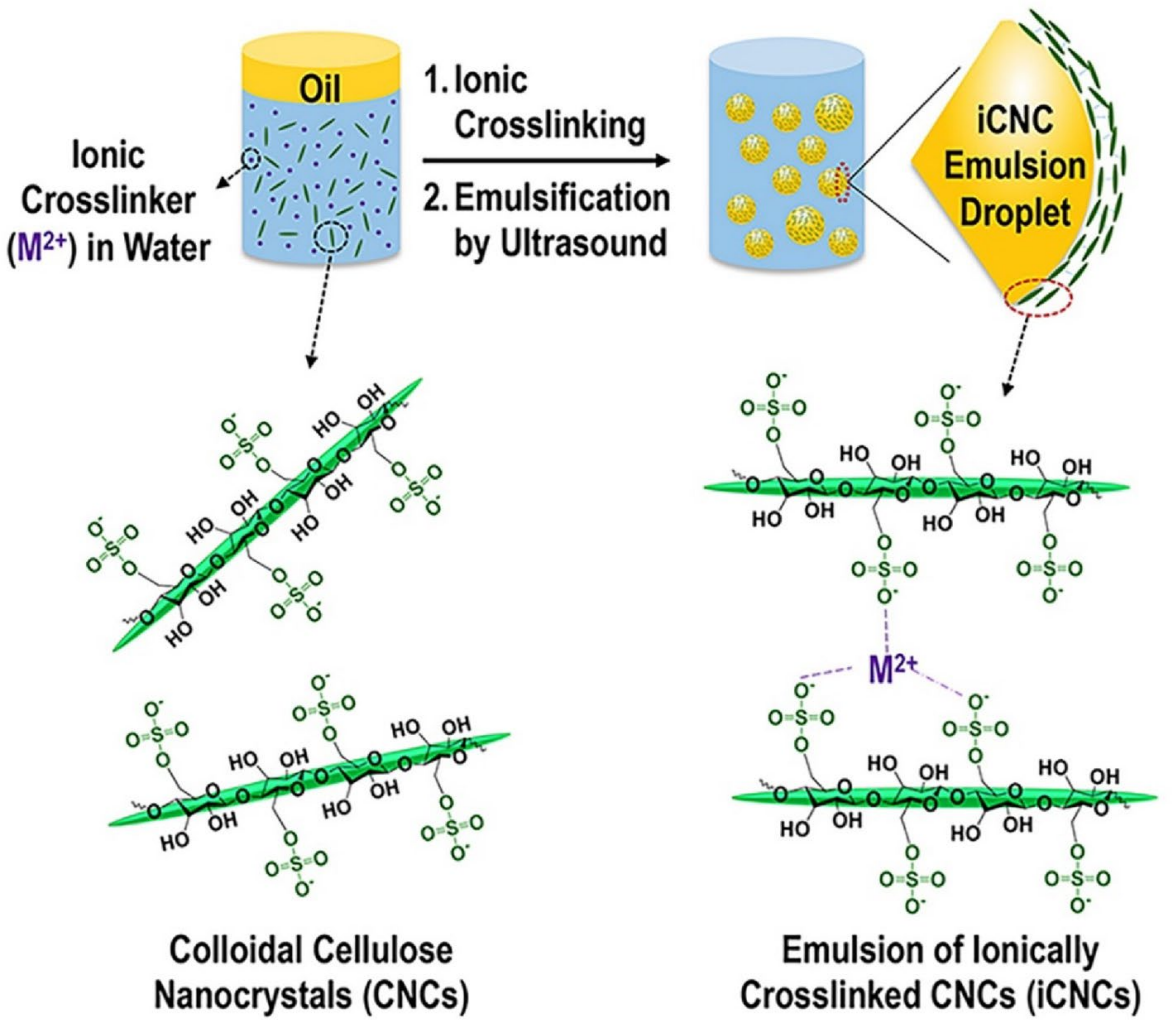
Schematic to illustrate the process and mechanism of droplet formation from the ionic crosslinking of CNCs to the emulsification of an oil in water.

Sulfated CNC suspensions were prepared by a 64% (w/v) sulfuric acid hydrolysis of microcrystalline cellulose from MP Biomedicals, LLC (Solon, OH, USA) at 45 °C for 40 min as previously reported^55,56^. The physicochemical characteristics^57,58^ of the prepared sulfated (i.e., –${{\text{SO}}}_{3}^{-}$) CNCs revealed a zeta potential (ZP) in the value range of –43.26 ± 2.11 mV at pH 6 with size of 117.14 ± 6.04 nm in length and 12.71 ± 2.67 nm in width, as imaged by atomic force microscopy (AFM) (see Supplementary Fig. S10). The aqueous phase consisted of varied concentrations (i.e., 5 mM, 10 mM, and 30 mM) of Mg(NO_3_)_2_ or Zn(NO_3_)_2_ (Sigma Aldrich, St Louis, MO, USA) as sources of divalent cations (i.e., ${{\text{Mg}}}^{2+}$ or ${{\text{Zn}}}^{2+}$) and sulfated CNCs [0.25%, 0.5% and 1% (w/v)]. Oleic acid (Sigma Aldrich) was the oil model used in this work. The ratio of water to oil was 90:10 (v/v) to prepare liquid like emulsions. The preparation of the o/w emulsion was done in two-steps, which involves (1) crosslinking sulfated CNCs in the aqueous phase with one of the divalent cations at room temperature (22ºC) and overnight incubation through electrostatic interaction, followed by (2) an ultrasonic emulsification using a Virtis Virsonic 100 probe ultrasonicator (SP Scientific, Warminster, PA, USA) for a total process time of 20 s with 2-s OFF and 3-s ON intervals at an intensity level of 50% (Fig. 9).

Before the ultrasonication process (i.e., process to emulsify oil in CNC), oil occupied the top layer of the sample, indicating a mixture of two immiscible liquids. After emulsification, o/w droplets were formed and emulsified progressively with increasing CNC and salt concentrations as ionically crosslinked CNCs (iCNCs) adsorb in between the oil and water surfaces to make uniform mixtures from partial to moderate to complete emulsions. A droplet test method^59^ was performed to determine that the emulsion type was that of an oil in water (see Supplementary Fig. S11).

### Light microscopy and visual observations

Imaging of each emulsion sample was performed using an inverted light microscope (Zeiss Axioskop 2, Carl Zeiss AG, Germany). Droplet size was determined within one day and at around one week of emulsion preparation, samples were examined at room temperature (22 °C), and micrographs of the emulsions were taken with a 20× objective (Carl Zeiss AG), which was powerful enough to image droplets with a wide area of view. Image processing was carried out using a public domain software ImageJ. For each emulsion, 10 μl of the solution was placed in a hole of a double-cavity microscope slide for visualization under the microscope.

Visual observations were used to validate the preparation of emulsions, and creaming index ($CI$) values were measured to evaluate emulsion stability over time, as well as the effect of CNC and salts on the formation of droplets. Additionally, visual observations were used to confirm the emulsion formation by simply monitoring the incremental miscibility of the oil and aqueous phase from each sample. The percent $CI$ was calculated as reported in previous work (Eq. 1)^60^ and express by:

1 $$CI={(H}_{C}/{H}_{T })\times 100$$

where ${H}_{C}$ and ${H}_{T}$ are the height of the cream and the total height of the dispersion contained in a cylindrical vial, respectively.

### Zeta potential (ZP)

A Zetasizer Nano ZSP ZEN 3600 (Malvern Instruments, UK) was used to assess the effect of surface charge on the formation of o/w emulsions at pH 6. The analyzer was set up at a wavelength of 633 nm in an electrophoretic light scattering mode with a detection fixed angle of 173 °C. Disposable folded capillary cells (DST1070) were used to contain all samples in solution. The temperature was set at room temperature (22 °C), and the samples were put to soak in the measuring cell for 120 s before starting measurements. The Smoluchowski model was used to determine the ZP values from the conversion of the electrophoretic mobility data to the surface charge of particles. Before each measurement, emulsions were 100 times diluted with deionized water. To determine ZP values, 15 cycles of ten measurements were averaged from electrophoretic mobility data points.

### Interfacial tension (IFT)

A goniometer OCA 15 (DataPhysics Instruments, Charlotte, NC, USA) was used at room temperature (22 °C) in a pendant drop method to evaluate the effect of the IFT on the formation of emulsion droplets. The instrument was equipped with an automated liquid dispenser for the release of droplets, a video camera for image acquisitions, and SCA20 software for image processing and droplet contour analyses. The equipment was calibrated with deionized water when IFT values were between 72.5 and 73 mN/m. After calibration, 10 µl of each emulsion solution was monitored every second from the video until stabilized, and results were recorded over 60 s in all treatments for comparison purposes. Measurements were performed in triplicates and the results were based on their average values with their respective standard deviations.

### Fourier transform infrared spectroscopy

Fourier transform infrared (FTIR) was performed to obtain spectra in absorption mode with wavelength values in the range of 4000–650 cm^−1^ using a PerkinElmer^®^ Frontier FTIR (Boston, MA, USA) equipped with mid-infrared (MIR)/attenuated total reflectance (ATR) to assess the effect of the surface chemistry on the formation of the CNC-based emulsion. The FTIR equipment allowed to differentiate surface chemistry based on sample treatments. All samples were dried in antistatic weighing boats for 72 h before analysis. In total, 40 scans per spectrum were performed at 4 cm^−1^ resolution to generate three spectra that were averaged for analysis.

### Rheology

Rheological analyses were done using a controlled strain Discovery Hybrid Rheometer (DHR-2) from TA Instruments (New Castle, DE, USA) to assess the stability and fluid behavior of the emulsion. A cone plate with 40 mm diameter and 1° angle was the measuring system. The fluid temperature at the Peltier Plate was maintained at 22 °C by an integrated Smart Swap® system. The rotational upper plate was used to transfer applied stress from the fluid to the bottom static plate and measurement was made with a 28 µm gap between the two plates. All rheological characterizations of emulsions were measured within 24 h after ultrasonication to assess the emulsion fluid behavior. A flow ramp mode at 1% strain was used to measure the apparent viscosity $\eta$ with shear rate ($\dot{\gamma }$) in the value range from 0.01 to 100 s^−1^. The evaluated emulsion was subjected to an oscillatory strain (Eq. 2) of angular frequency, $\omega =2\pi f$ [rad/s], which is defined by a period ($t$) given by the frequency ($f$). The oscillation strain function $\gamma (t)$ was expressed by:

2 $$\gamma (t)={\gamma }_{o}{\text{sin}}\left(\omega t\right)$$

Samples were subjected to oscillation shear stress over a small amplitude (${\gamma }_{o}$), allowing the measurement of the of storage (${G}{\prime}\left(\omega \right)$) and loss moduli (${G}^{{\prime}{\prime}}\left(\omega \right)$) to assess the microstructural behavior of formed emulsions.

### Statistical analyses

Results were shown as means ± the standard deviation (n = 4) and any difference in the data were statistically significant when *p* < 0.05 using JMP^®^ software (SAS Institute Inc., Cary, NC, USA).

### Supplementary Information

Supplementary Figures.

## Data Availability

All data used and discussed in this study are included in this article and supplementary information.
