# Supplementary material for: Ionically crosslinked cellulose nanocrystals by metal nitrates for the preparation of stable emulsions with tunable interface properties
Source: Sci Rep. 2023 Dec 7;13:21630. doi: 10.1038/s41598-023-48703-3 (PMC10703815; doi:10.1038/s41598-023-48703-3)
Supplement: Supplementary file 1 — Supplementary Figures. [file 41598_2023_48703_MOESM1_ESM.pdf]

Supplementary Information

Ionically crosslinked cellulose nanocrystals by metal nitrates for the preparation of stable emulsions with tunable interface properties

Joseph Batta-Mpouma[1,2], Gurshagan Kandhola[1,3] & Jin-Woo Kim[1,2,3]✉

[1]Bio/Nano Technology Group, Institute for Nanoscience & Engineering, University of Arkansas, Fayetteville, Arkansas 72701, United States
[2]Materials Science & Engineering Program, University of Arkansas, Fayetteville, Arkansas 72701, United States
[3]Department of Biological & Agricultural Engineering, University of Arkansas, Fayetteville, Arkansas 72701, United States

✉e-mail: jwkim@uark.edu

Number of pages: 12

Number of figures: 11

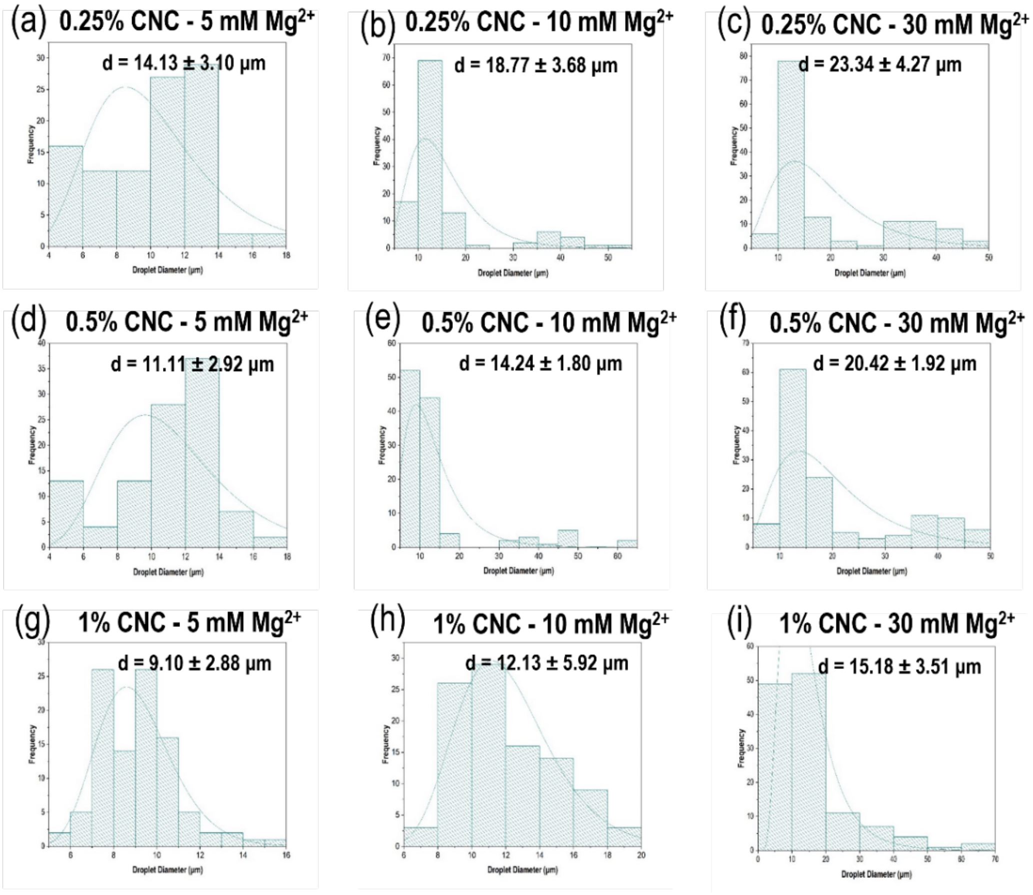

Figure S1. Size distributions showing droplet diameters within 24 h for emulsions made at varied concentrations of CNCs and $Mg(NO_3)_2$.

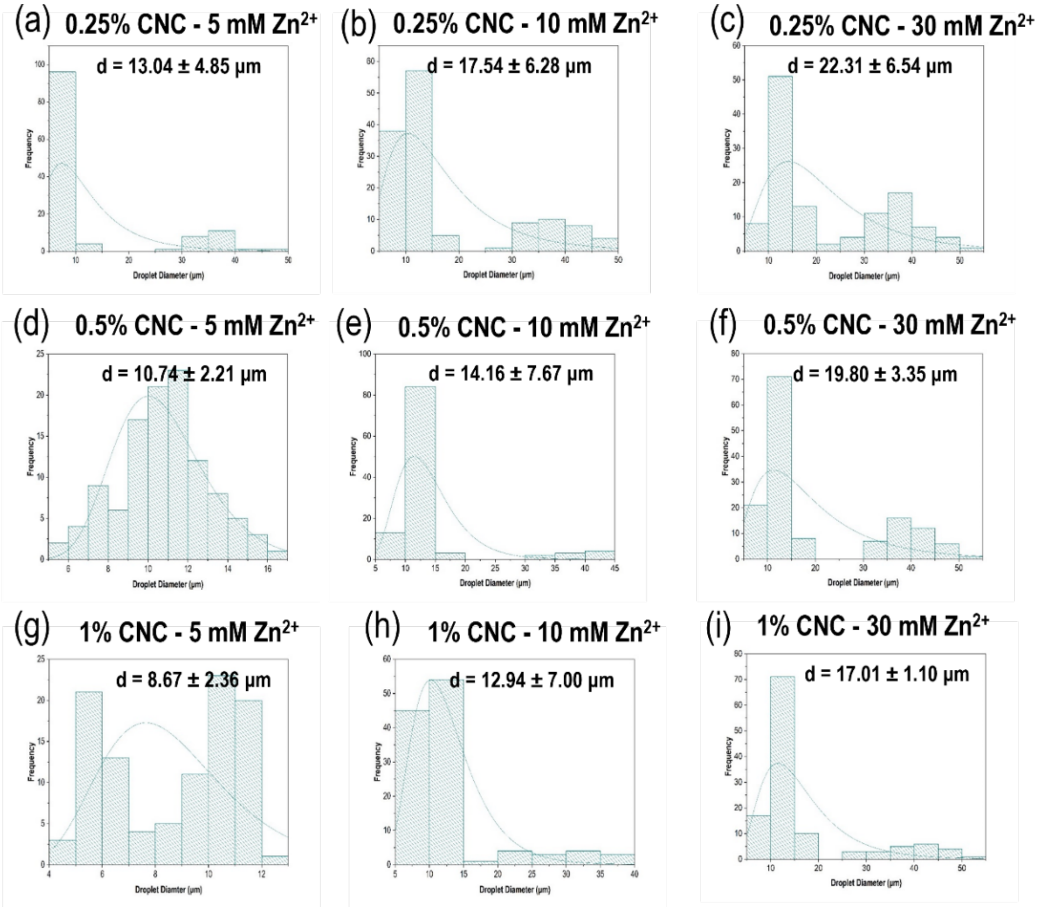

Figure S2. Size distributions showing droplet diameters within 24 h for emulsions made at varied concentrations of CNCs and $Zn(NO_3)_2$.

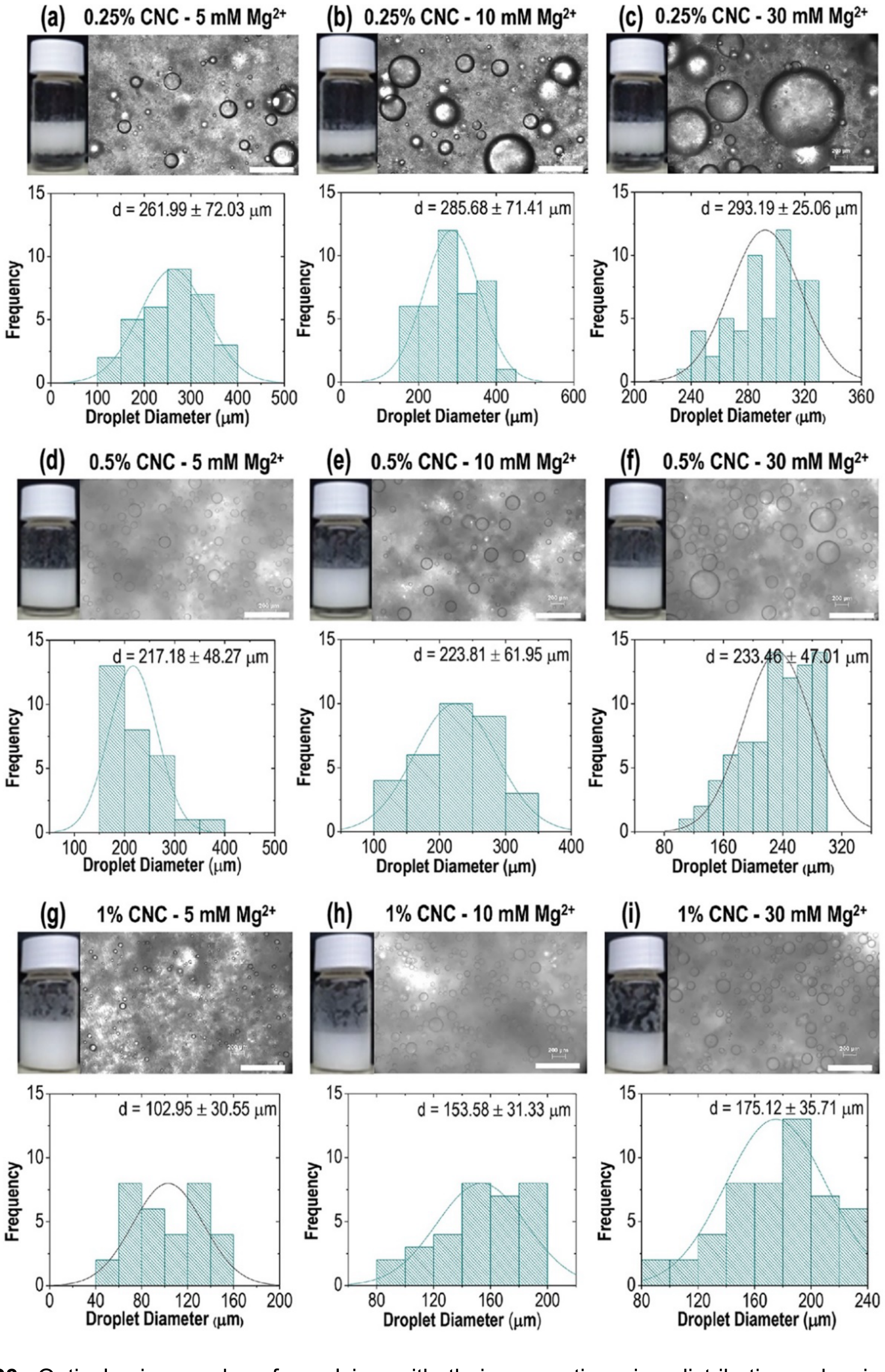

Figure S3. Optical micrographs of emulsion with their respective size distributions showing droplet diameters at around 7 days of preparation for emulsions made at varied concentrations of CNCs and $Mg(NO_3)_2$. Scale bars, 200 μm.

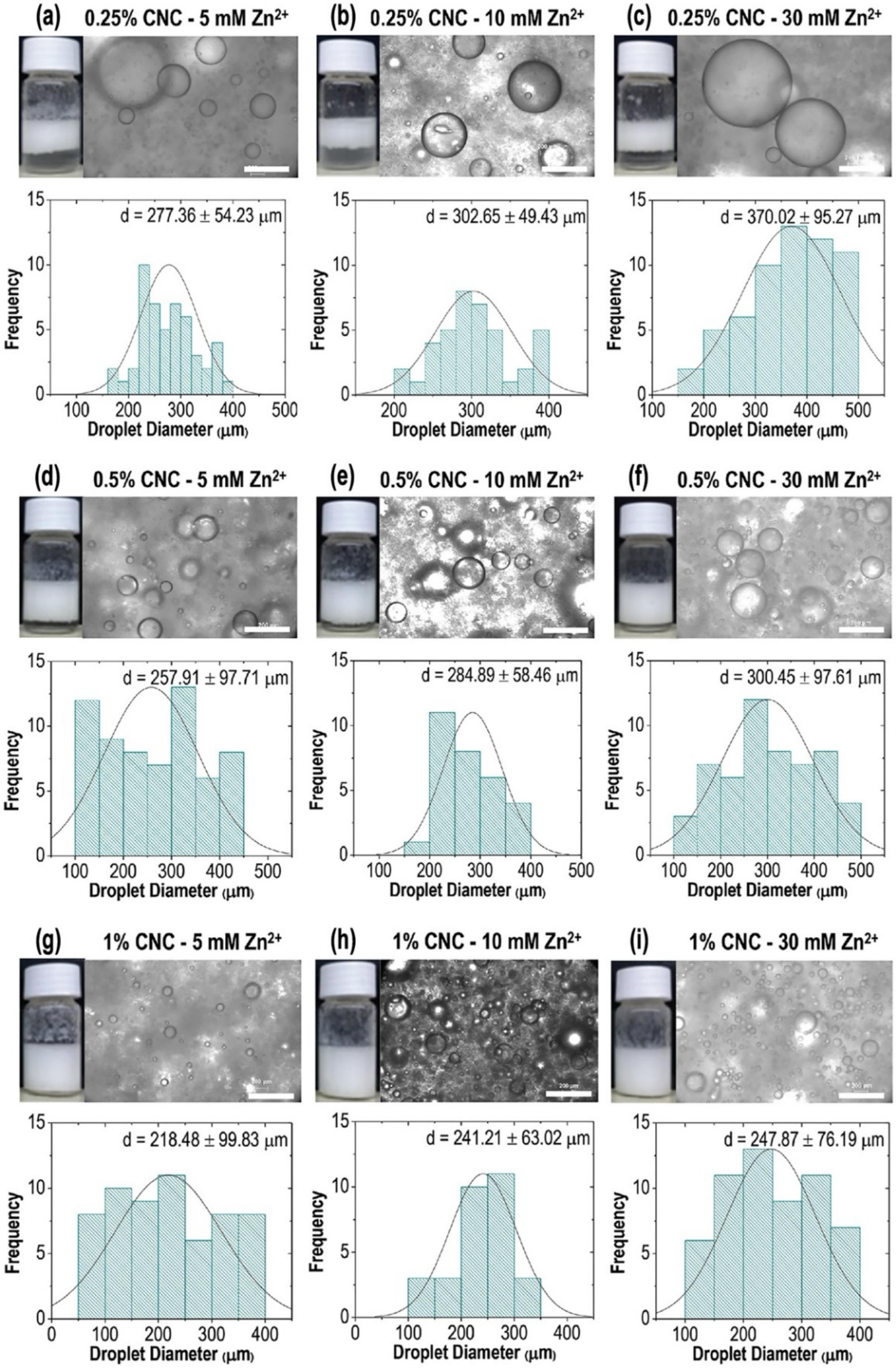

Figure S4. Optical micrographs of emulsion with their respective size distributions showing droplet diameters at around 7 days of preparation for emulsions made at varied concentrations of CNCs and $Zn(NO_3)_2$. Scale bars, 200 μm.

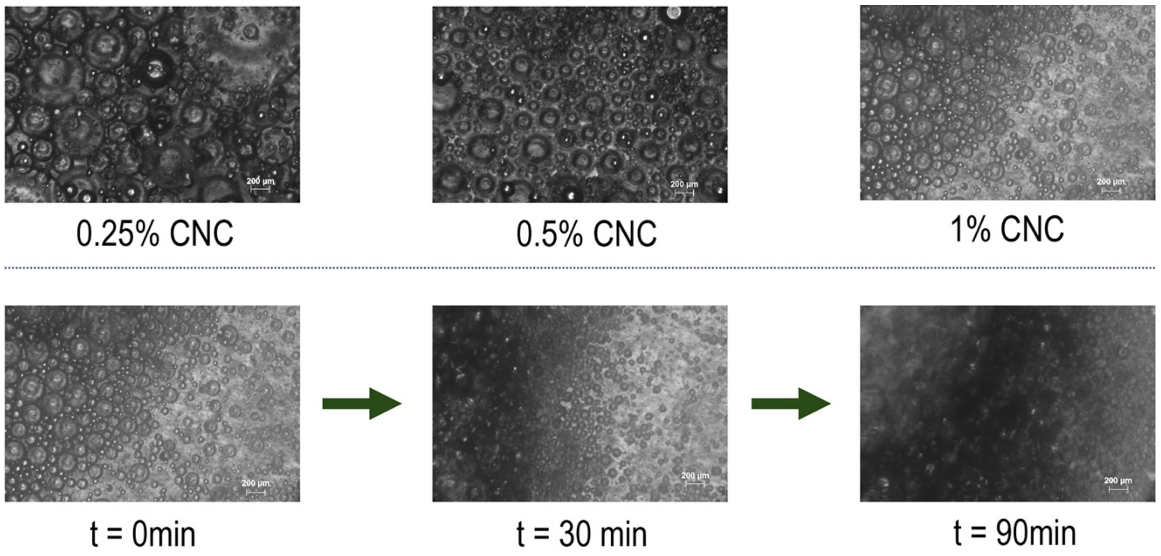

Figure S5. (Top) Light microscopy showing coalescence of emulsion droplets at different CNC concentrations (w/v) without prior ionic crosslinking, and (bottom) light microscopy showing disintegration of the droplets over time (90 min) for o/w emulsion with 1% CNC and no prior ionic crosslinking with divalent salts. Scale bars, 200 μm.

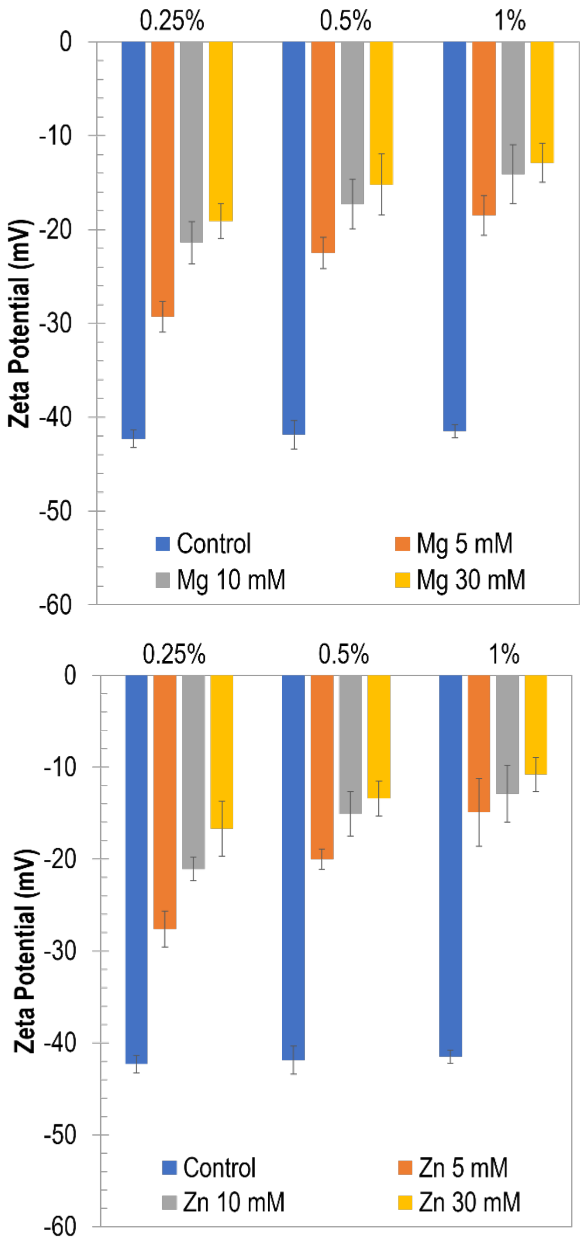

Figure S6. Zeta potential of ionically crosslinked CNCs (iCNCs) as a function of CNC concentration at varied concentrations of (a) $Mg(NO_3)_2$ and (b) $Zn(NO_3)_2$ to show the effect of surface charge on crosslinking.

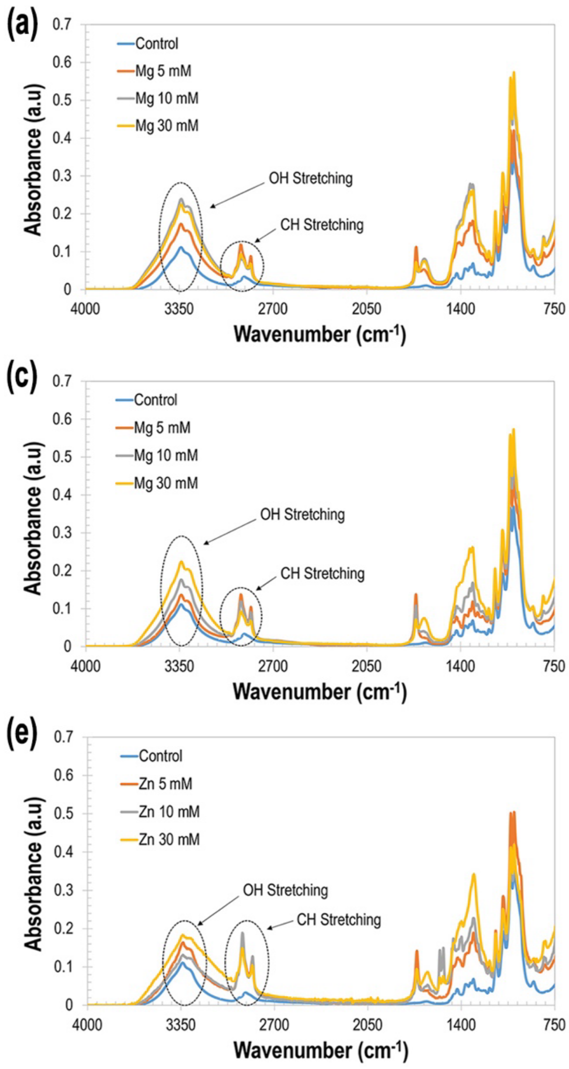

Figure S7. Fourier transform infrared (FT-IR) as a function of wavenumber at varied concentrations of divalent salts for iCNCs to study the effect of particle adsorption at the o/w interface: (a) 0.25% (w/v) and $Mg(NO_3)_2$, (b) 0.5% and $Mg(NO_3)_2$, (c) 1% and $Mg(NO_3)_2$, (d) 0.25% and $Zn(NO_3)_2$, (e) 0.5% and $Zn(NO_3)_2$, and (f) 1% and $Zn(NO_3)_2$. Note: This figure shows the entire spectra of the FT-IR analyses.

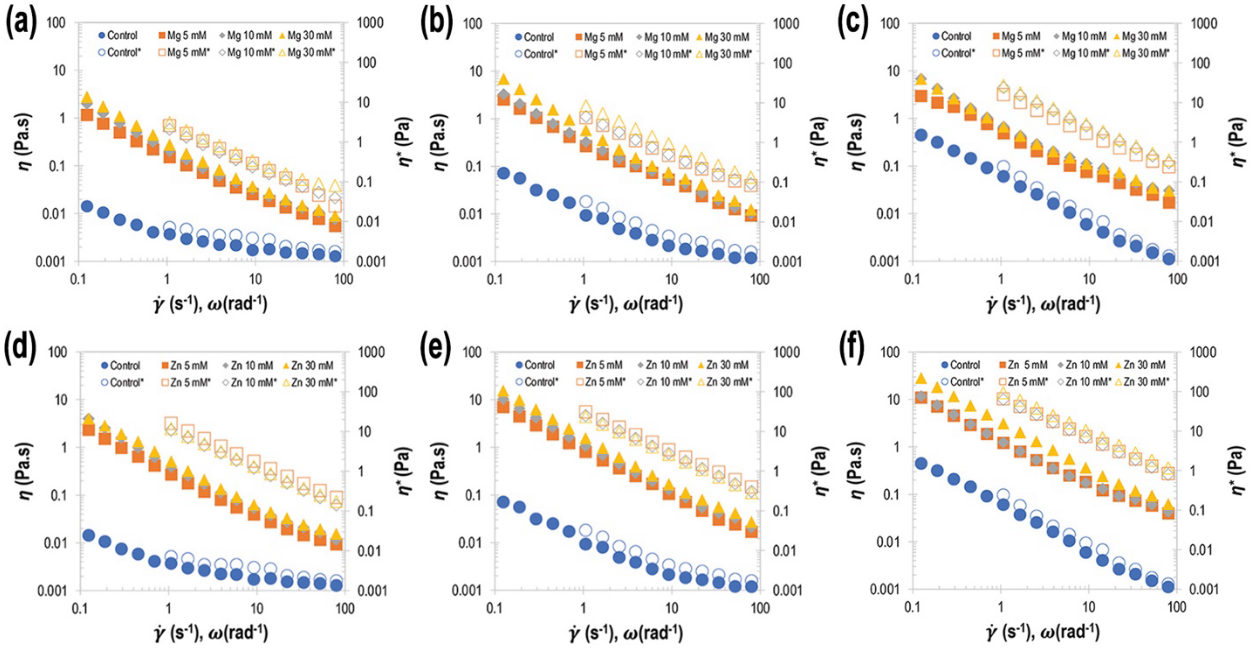

Figure S8. Cox-Merz plots at varied concentrations of CNCs and divalent salts: (a) 0.25% (w/v) and $Mg(NO_3)_2$, (b) 0.5% and $Mg(NO_3)_2$, (c) 1% and $Mg(NO_3)_2$, (d) 0.25% and $Zn(NO_3)_2$, (e) 0.5% and $Zn(NO_3)_2$, and (f) 1% and $Zn(NO_3)_2$. Open symbols: Complex viscosity (η*) vs steady shear rate ($\dot{\gamma}$) or angular frequency (ω). Closed symbols: Apparent viscosity (η) vs $\dot{\gamma}$ or ω.

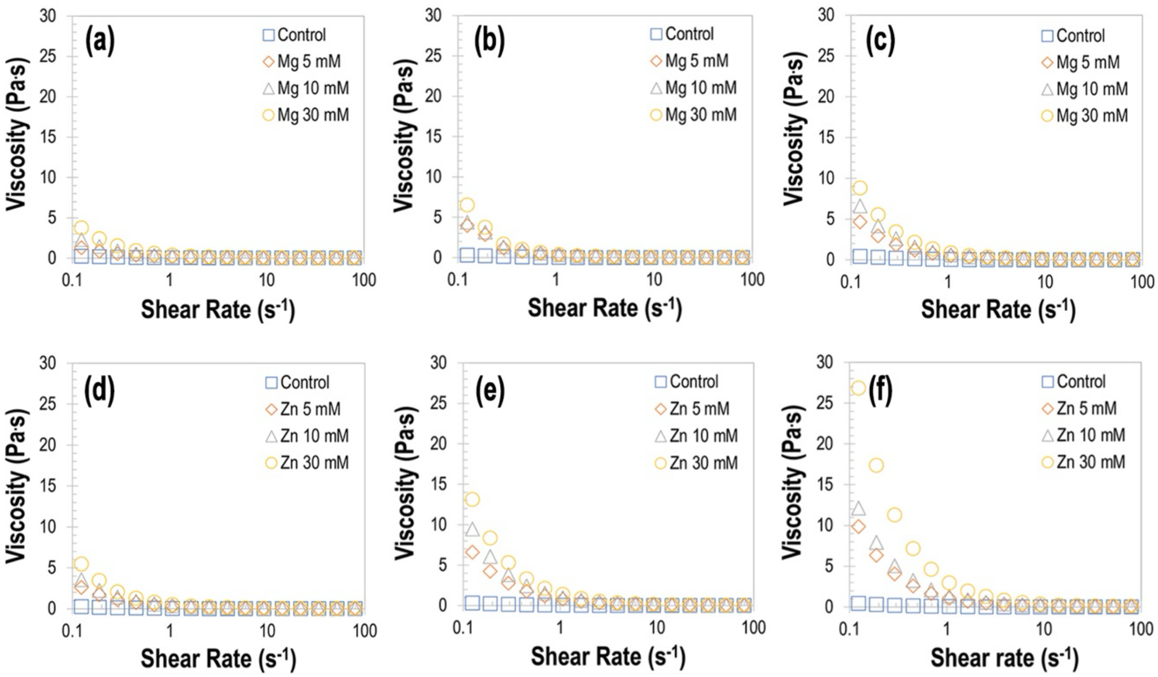

Figure S9. Shear viscosity as a function of shear rate at varied concentrations of divalent salts for iCNCs to study the effect of particle adsorption at the o/w interface: (a) 0.25% (w/v) and $Mg(NO_3)_2$, (b) 0.5% and $Mg(NO_3)_2$, (c) 1% and $Mg(NO_3)_2$, (d) 0.25% and $Zn(NO_3)_2$, (e) 0.5% and $Zn(NO_3)_2$, and (f) 1% and $Zn(NO_3)_2$.

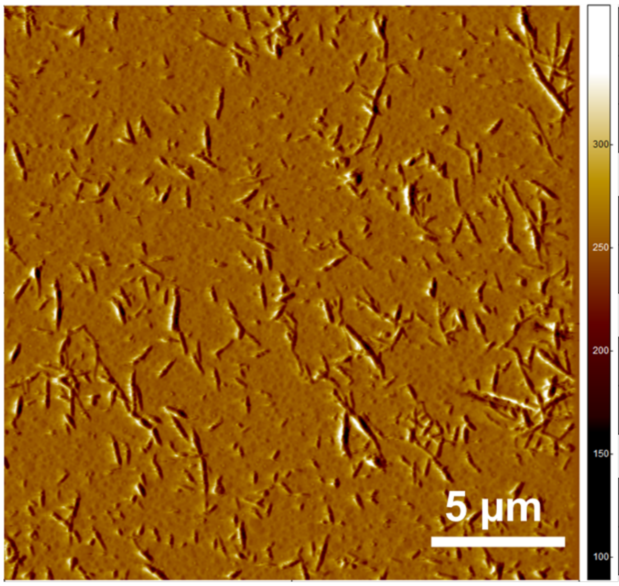

AFM Size (nm)
L: 117.14 ± 6.04
W: 12.71 ± 2.67

Hydrodynamic Size (nm)
128.62 ± 3.71

Zeta Potential (mV)
– 43.26 ± 2.11

Poly Dispersity Index
0.49 ± 0.04

Figure S10. Physicochemical characteristics of colloidal CNC.

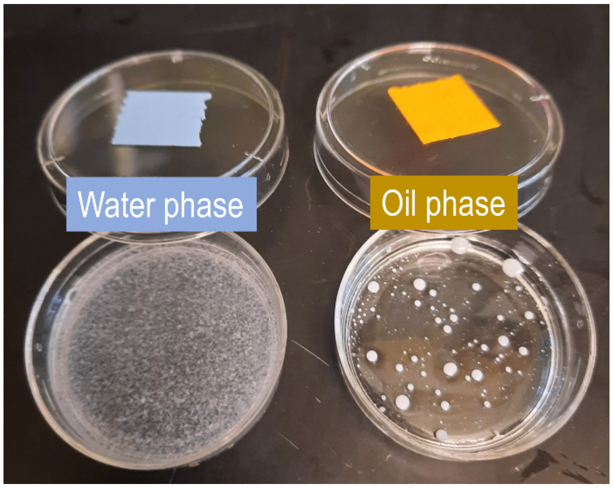

Figure S11. Droplet test method to determine emulsion type (i.e., oil-in-water), using ionically crosslinked CNC (1% w/v) in $Mg(NO_3)_2$ (30 mM).
